# Modelling the relationship between the WOMAC osteoarthritis index and EQ-5D

**DOI:** 10.1186/1477-7525-12-37

**Published:** 2014-03-12

**Authors:** Allan Wailoo, Monica Hernandez Alava, Antonio Escobar Martinez

**Affiliations:** 1 Health Economics and Decision Science, School of Health and Related Research (ScHARR), University of Sheffield, Sheffield, UK; 2 Red de Investigación en Servicios de Salud y Cronicidad (REDISSEC), Hospital Universitario Basurto, Bilbao, Spain

**Keywords:** EQ-5D, WOMAC, Mapping, Osteoarthritis, Statistical methods

## Abstract

**Objective:**

Economic evaluation typically is conducted using health state utilities to estimate treatment benefits. However, such outcomes are often missing from studies of clinical effectiveness. This study aims to bridge that gap by providing appropriate methods to link values from the Western Ontario and McMaster Universities Osteoarthritis Index (WOMAC) to the EQ-5D utility instrument.

**Method:**

Patients from a large registry of Spanish patients (n = 7072 observations) with knee or hip osteoarthritis who completed both WOMAC and EQ-5D was used. A mixture model approach was used based on distributions bespoke to the EQ-5D UK value set to estimate EQ-5D as a function of WOMAC pain, stiffness and function subscores.

**Results:**

A five class mixture model provides very close fit to the observed data at all levels of disease severity. The overall mean (0.542 vs 0.542), median (0.620 vs 0.636) and the percentage of observations at full health (15 vs 14.8) were very similar between the observed data and the estimated model respectively. Stiffness has limited relationship to EQ-5D, whereas functional disability and pain are strong predictors.

**Conclusion:**

EQ-5D can be reliably estimated from WOMAC subscale scores without any systematic bias using these results.

## Background

Osteoarthritis is one of the leading causes of pain and limitation in function, typically affecting the hip, knee and small hand joints. It is the most common form of arthritis and is characterised by extreme variability in clinical presentation, rate of disease progression and patient outcomes.

One of the most widely reported outcome measures in Knee and Hip osteoarthritis is the Western Ontario and McMaster Universities Osteoarthritis Index (WOMAC) [1]. WOMAC comprises 24 questions covering the dimensions of pain, stiffness and physical functioning. With substantial evidence of psychometric validity it is in widespread use in clinical studies designed to assess the efficacy of technologies in this disease area.

However, WOMAC is not a tool suitable for use in economic evaluation. In particular, whilst analysts typically seek to evaluate interventions in terms of costs and Quality Adjusted Life Years (QALYs), the scores from the WOMAC instrument are not appropriate since they do not provide either a cardinal or preference based scale. Generic instruments that would facilitate the calculation of benefits in terms of QALYs, such as the EQ-5D, Health Utilities Index Mark 3 and SF-6D, have rarely been included in clinical studies. There is therefore often a gap in the evidence.

One method of bridging this evidence gap is by using statistical methods to estimate the relationship between clinical outcomes, such as WOMAC, and measures suitable for use in economic evaluation, using separate datasets of patients that have completed both instruments. This allows the benefits of treatment to be converted to an appropriate scale for economic evaluation. This practice has been variously labelled “mapping”, “cross-walking” and “transfer to utility”.

However, utility data exhibit peculiar characteristics that raise statistical challenges: they are limited at full health (1) and tend to have a mass of observations at that upper bound, they are limited below at the worst health state described. EQ-5D tends to be bi or tri-modal with a gap between full health and the next feasible health state. Standard regression methods are not suited to data with such distributions and the resulting bias has been demonstrated in many disease areas [2,3] and is evidenced in the published evidence on WOMAC and osteoarthritis [4,5]. This bias leads to the systematic undervaluing of the benefits of health care technologies in economic evaluations, and ultimately to inappropriate decisions for patients and their treating clinicians.

**Figure 1:**
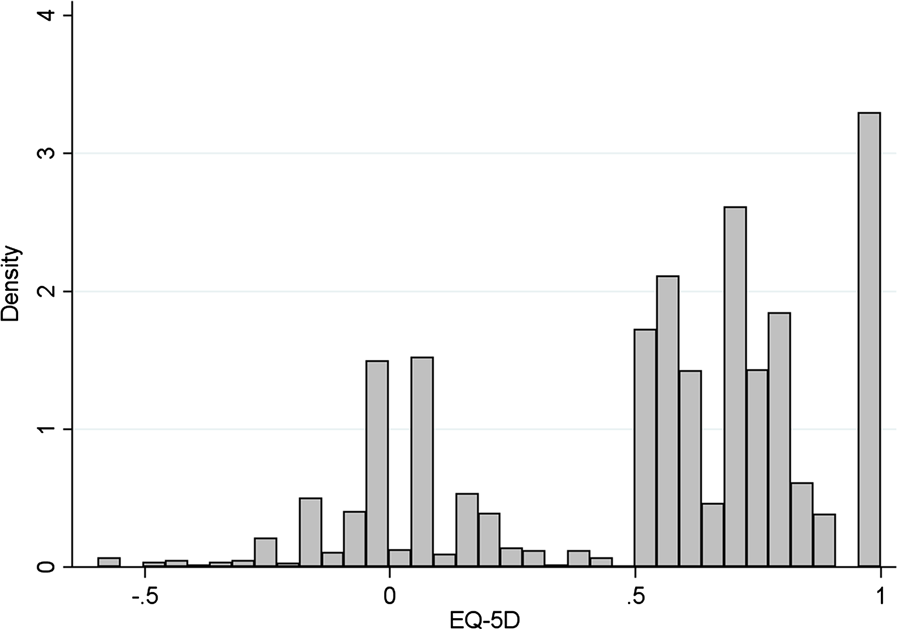
Distribution of EQ-5D summary scores.

Barton et al. [5] (n = 348), Xie et al. [6] (n = 258), Marshall et al. [4] and Grootendorst et al. [7] (n = 2186) all use linear regression to estimate health state utilities (either based on the EQ-5D or HUI-3) from the WOMAC instrument. Barton et al. [5] (Figure 1) and Marshall et al. [4] (Figures 1a and b), which takes model results from Grootendorst et al. [7], present results that show how the fit of the statistical model varies across the range of disease severity and, as expected a priori, there is clear evidence of systematic bias: the models overpredict utility values for patients with relatively severe disease and underpredict values for those patients at higher levels of health.

New methods have been developed specifically for modelling EQ-5D and been demonstrated to overcome the limitations of standard methods in other disease areas [8]. This paper reports on the application of such methods to modelling EQ-5D from WOMAC and other explanatory variables using data from patients with osteoarthritis of the hip or knee.

## Methods

### Dataset

A total of 1768 patients were recruited to this registry study conducted in 15 hospitals from three regions of Spain: 3 in Andalusia, 3 in the Canary Islands, and 9 in the Basque Country. The Institutional Review Boards of each hospital approved the study.

Consecutive patients scheduled to undergo primary joint replacement surgery due to knee or hip osteoarthritis in one of the participating hospitals between October 2005 and October 2006 and who received postoperative management in the hospitals were eligible for the study. Patients with cancer or severe organic or psychiatric diseases were excluded because these conditions could prevent them from completing all the questionnaires included in the study.

All patients were sent a letter informing them about the study and asking for their voluntary participation. Questionnaires were mailed to each patient at baseline prior to surgery, 3, 6 and 12 months after surgery. Reminder letters were sent 15 days after each mailing to patients who had not replied promptly. The baseline questionnaire included items about expectations for the operation, along with the SF-12, WOMAC, and EQ-5D-3L questionnaires, plus questions requesting sociodemographic information like gender, age, hospital, weight and height (for the calculation of body mass index [BMI]).

A total of 7072 observations were obtained.

We applied the EQ-5D UK tariff for use in this analysis. The Western Ontario and McMaster Universities Osteoarthritis Index (WOMAC) is one of the most widely reported outcome measured in clinical studies of osteoarthritis. It comprises 24 questions with responses to each given on a Likert scale: none (0), mild (1), moderate (2), severe (3) or extreme (4) problems. It is possible to construct a single index score between 0–96 by summing the scores to each question or one may consider subscale scores based on pain (5 questions yielding a range of 0–20), stiffness (2 questions range 0–8) and functional impairment (17 questions range 0–68).

### Statistical methods

Health state utility values in general, and the EQ-5D instrument in particular, are characterised by several features that render standard statistical models redundant. Feasible values are limited both above and below at full health and the worst health state described by the instrument. There tend to be a large number of individuals that indicate they are at full health. There is a relatively large gap from full health to the next feasible value (0.883). The typical distribution of EQ-5D tends to be tri-modal with distinctly non-normal elements within each section. In this situation it is obvious that standard multiple regression models, which assume conditional normality, are inappropriate. There now exists a battery of studies that consistently demonstrate this to be the case in a range of disease areas. Linear regression leads to estimates that are consistently biased downwards when considering the value of change in disease level on the EQ-5D scale.

We have previously developed a method specifically designed to produce appropriate estimates of EQ-5D from clinical and other predictors. It has been demonstrated to overcome the limitations of other commonly used direct and indirect methods in several different settings and full details of the model are provided elsewhere [8,9]. We apply this method here in order to estimate EQ-5D from WOMAC responses, incorporating other socio-demographic and clinical covariate information.

The method applied here uses multiple regression with two novel features. First, we use mixture models which identify a number of different classes within the data which when combined form a new distribution. Whilst the individual classes may estimate EQ-5D as a function of WOMAC and other variables based on standard distributional forms, when these are combined they can form very complex, non standard distributions such as the distribution of EQ-5D. Whilst the relationship between EQ-5D and each of the explanatory variables will differ for each class it is not the identification of different classes that leads us to use this framework, though this may be a valuable insight. It is the flexibility of the modelling framework that makes it particularly useful for modelling EQ-5D values ([10]). The overall estimate of EQ-5D, predicted from any set of WOMAC pain, stiffness and disability scores, is a weighted function of these individual components.

The second novel feature of the analysis is that, in this case, instead of basing each separate class of the mixture on a standard normal distribution, we base it instead on a distribution specific to the characteristics of EQ-5D, namely, limited above at full health (1), below at −0.594 and adjusted to reflect the gap in feasible values between 1 and 0.883.

Explanatory variables may enter the model in two ways: either as predictors of the relationship with EQ-5D within each of the individual classes, as in standard regression, or as predictors of component membership. We compared several different variants of using the explanatory variables in these two ways. Within each class we adopted a consistent approach to the inclusion of variables, opting not to exclude non-significant terms on an individual basis.

## Results

Table 1 displays the characteristics of patients in the dataset at baseline assessment and shows that there is substantial variability across patients. Mean age is 69 years (sd 9.8) with a range of 25 to 100 years. The mean BMI of 29.7 (sd 4.7) is in the “overweight” category but patients span all categories, from “underweight” to “obese”.

**Table 1:** Characteristics of the patient sample

**Variable**	**Obs**	**Mean**	**SD**	**Min**	**Max**
Age (yrs)	1593	69.14	9.81	25.64	100.04
Body Mass Index	1455	29.65	4.67	14.17	50.22
EQ-5D	1520	0.29	0.35	−0.594	1
WOMAC pain scale	1660	11.37	3.78	0	20
WOMAC stiffness scale	1657	4.70	1.95	0	8
WOMAC function scale	1664	43.85	11.83	0	68
Proportion - Osteoporosis of the hip	1768	0.61	0.49		
Proportion - Male	1764	0.39	0.49		

WOMAC scale scores indicate that patients are of moderate severity on average at baseline, as expected from a hospital recruited cohort. Similarly mean EQ-5D of 0.29 (sd 0.35) indicates a significant reduction in quality of life with patients spanning the entire feasible range for the scale (−0.594 to 1). This is reflected in Figure 1 which provides a histogram of EQ-5D scores collected over the 4 time points. This illustrates a distribution with the classic characteristics of EQ-5D data: there is a mass of observations at full health (1), a gap between these observations and the rest, and at least two additional modes. In total there are 598 observations, 8.5% of the sample, at this upper limit.

We considered models with 3, 4 and 5 components. The preferred model was a mixture of five components on the basis of summary measures of fit (MAE, RMSE, AIC, BIC) compared to standard linear regression and mixtures with different numbers of components. Estimates from the preferred model are shown in Table 2. Within each class, we found that EQ-5D was best explained by linear terms for WOMAC pain, stiffness and function subscores. Pain was significantly and substantially negatively related to EQ-5D in class 2. In most other classes the effect was not significant, except class 3 where a positive relationship was identified. Stiffness has only a minor impact on predicting EQ-5D within any of the classes: class 4 has a small negative relationship (p < 0.1). Function has a much stronger relationship with EQ-5D within all the classes. Coefficients are negative for all classes, this is statistically significant at the 10% level in 4 classes and almost significant in the remaining class (class 2). The effect is greatest in class 3.

**Table 2:** Parameter estimates from the 5 class mixture model

		**Class 1**			**Class 2**			**Class 3**			**Class 4**			**Class 5**		
	**Parameter**	***p-value***			***p-value***			***p-value***			***p-value***			***p-value***		
*Within class*	Pain/10	0.0166	*0.260*		−0.2357	*0.000*	***	0.1475	*0.000*	***	0.001	*0.285*		0.0116	*0.600*	
	Stiffness/10	0.0118	*0.583*		−0.0287	*0.760*		−0.0182	*0.662*		−0.0029	*0.057*	*	0.0037	*0.914*	
	Function/10	−0.0356	*0.000*	***	−0.0274	*0.103*		−0.07	*0.000*	***	−0.0003	*0.087*	*	−0.0403	*0.000*	***
	Variance	0.0024	*0.000*	***	0.0406	*0.000*	***	0.0047	*0.001*	***	0	*0.000*	***	0.0058	*0.002*	***
*Random effects terms*	Intercept	0.7636	*0.000*	***	0.3762	*0.000*	***	0.9662	*0.000*	***	0.555	*0.000*	***	0.2118	*0.000*	***
	Age^1^	0.005	*0.000*	***												
	Male	0.0083	*0.000*	***												
	Variance	0.0016	*0.000*	***												
*Class probabilities*	Intercept	8.8821	*0.000*	***	7.0881	*0.000*	***	11.4076	*0.000*	***	5.1497	*0.0004*	***			
	Pain/10	−5.1772	*0.006*	***	−7.9363	*0.000*	***	−13.4885	*0.000*	***	−6.4654	*0.0006*	***			
	Stiffness/10	−1.0227	*0.625*		−6.1566	*0.004*	***	−0.7275	*0.751*		−1.0918	*0.6023*				
	Function/10	−1.3706	*0.026*	**	−0.9434	*0.162*		−2.0612	*0.002*	***	0.37	*0.5729*				
	(Pain/10)^2^	1.2266	*0.114*		2.3464	*0.001*	***	4.5149	*0.000*	***	1.4951	*0.0568*	*			
	(Stiffness/10)^2^	−1.158	*0.604*		3.7049	*0.156*		−1.483	*0.570*		−1.3521	*0.5465*				
	(Function/10)^2^	0.0797	*0.286*		0.1946	*0.008*	***	0.2545	*0.001*	***	−0.0626	*0.4179*				
*EQ-5D estimates by class*	Mean	0.678			0.124			0.881			0.557			0.106		
	Median	0.679			0.129			0.865			0.557			0.107		
	Min	0.307			−0.594			0.335			0.382			−0.377		
	Max	1			1			1			0.748			0.598		
	Proportion of ones	0.004			0.001			0.431			0.000			0.000		
	Prob of class membership	0.333			0.109			0.240			0.146			0.172		

Notes: ^1^Age = (age-mean age in sample)/10. Mean sample age is 69 years.

*: P<0.1, **: P<0.05, ***: P<0.01.

Pain, stiffness, function and their quadratic terms were entered as predictors of class membership. Estimates are also shown in Table 2. The probability of class 5 is 1 minus the sum of the probability of being in classes 1 to 4. Pain and function are important predictors of class membership whilst stiffness is statistically significant only as a determinant of class 2 membership.

Differences both in the determinants of class membership and the impact of explanatory variables within each class are partly due to the fact that each class is centred around different elements of the disease severity spectrum. The lower element of Table 2 provides statistics describing the location and spread of each class. Classes 2 and 5 are centred around the most severe patients with mean EQ-5D of approximately 0.1. This peak in the overall distribution of EQ-5D scores is clearly visible in Figure 1. Class 2 has a much greater variance however, spanning the entire feasible range of EQ-5D (−0.594 to 1). Note that because the model is designed bespoke to the features of EQ-5D it does not generate values outside the feasible range. Classes 1, 3 and 4 jointly reflect the peaks in the distribution seen around 0.7 and 1. Class 1 is the largest of the groups and has a mean EQ-5D of 0.68. Class 3 has a mean of 0.88 (the upper limit of EQ-5D values below full health) but is the only class that contributes a substantial proportion of full health observations.

Table 3 illustrates that for the mixture model overall, the mean value is virtually identical to the observed data. Similarly, the range of the data is equivalent to the data, by design. For the sake of comparison the liner regression equivalent is provided. The mean is quite close to the data for this model but other values illustrate how the model assumptions of conditional normality lead to poorer reflection of the overall distribution. In particular, the model predicts values that substantially exceed the feasible EQ-5D range, both above full health and below the “pits” state.

**Table 3:** Summary measures of performance

	**Data**	**Mixture model**	**Linear model**
Mean	0.5422	0.5421	0.5402
Median	0.620	0.636	0.552
SD	0.363	0.361	0.361
Minimum	−0.594	−0.594	−0.946
Maximum	1	1	1.942
Proportion of ones	15.02	14.81	1.121^*^

^*^The linear model is fully continuous. We have reported the proportion of values >0.99 and < 1.01.

**Figure 2:**
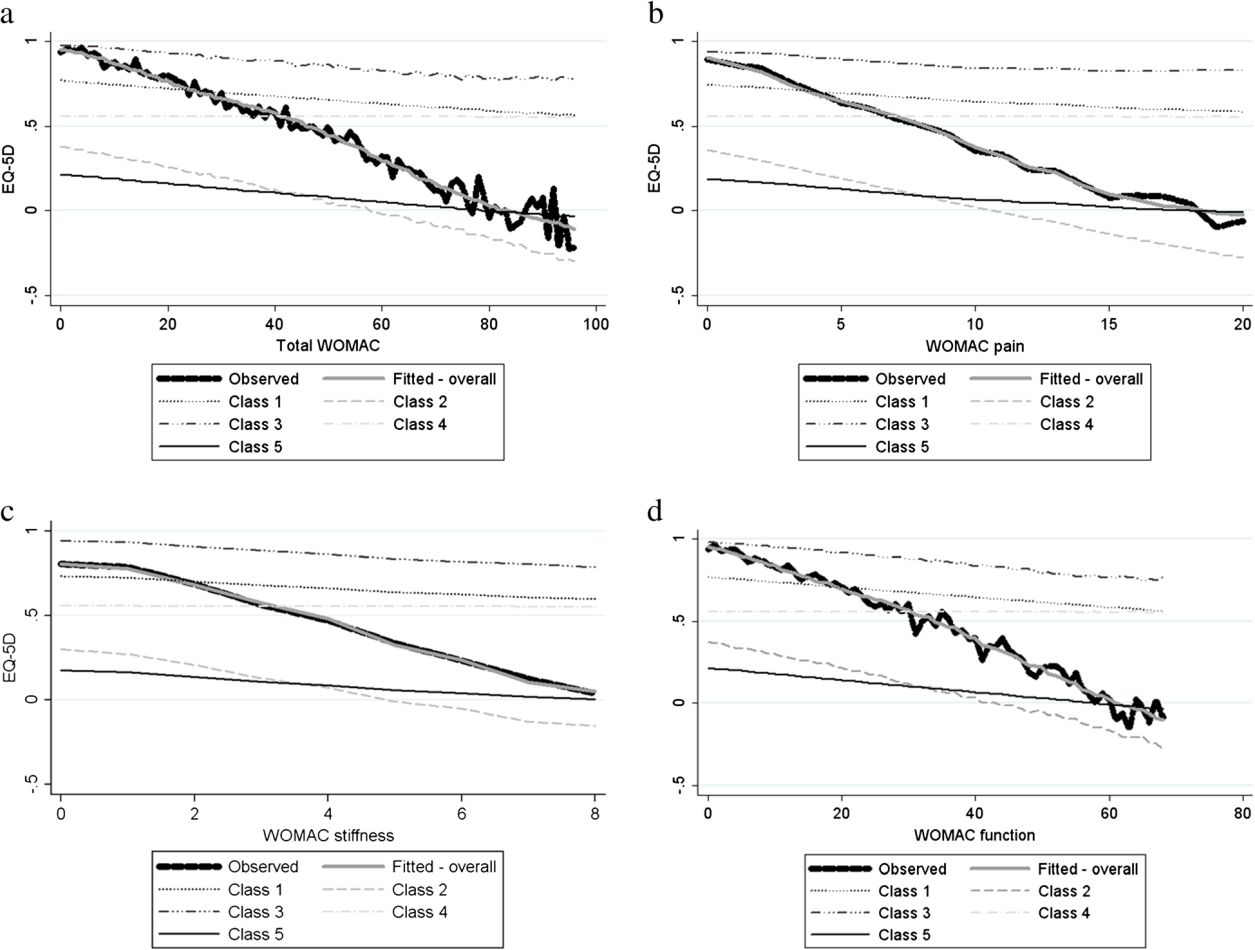
Mean EQ-5D by mean a) total WOMAC, b) WOMAC pain, c) WOMAC stiffness and d) WOMAC function, overall and by individual class.

Figure 2 compares the mean fitted EQ-5D values from the mixture model against the mean EQ-5D across the range of WOMAC disease severity for the summary score (Figure 2a), pain subscale (Figure 2b), stiffness subscale (Figure 2c) and function subscale (Figure 2d). The plots illustrate that the expected values generated from the statistical model fit the data extremely closely across the entire range of disease severity, however severity is defined. There is no evidence of systematic bias in the model fit.

The figures also illustrate the expected values for the individual classes. There is clear variation in the slopes for each class. Class 4 is the least responsive to changes in disease severity whilst class 2 is the most responsive. Note that this is not simply a reflection of the within class coefficients since patients with greater levels of disease severity on one measure also tend to have greater levels of impairment on other measures. None of the slopes are as steep as for the combined relationship which is formed as the weighted average of the five classes with the weights themselves a function of disease severity. The overall slope at low levels of impairment is formed predominantly from classes 1 and 3, whereas at the most severe levels of impairment it is formed from classes 2 and 5.

## Discussion

Whilst several previous studies have been published that look at the relationship between some measure of health utility and the WOMAC instrument, they are all based on linear regression models (Xie et al. [6] include a comparison with the Censored Least Absolute Deviations (CLAD) model). Theoretically these are unlikely to be appropriate, have been shown to be biased in many other disease areas, and also appear to be biased in the osteoarthritis setting [4,5]. Studies have also been based on relatively small samples of patients.

In this paper we have applied a method based on mixture models that suffers from none of these problems, using data of over 7,000 patient observations that cover the full spectrum of disease. It therefore provides an appropriate method to express treatment benefits from clinical studies which use the WOMAC as an outcome measure in terms of QALYs. This link between clinical and economic outcomes is increasingly important for reimbursement authorities. This is therefore not just of academic interest. Patients and their treating clinicians are directly affected by the methods used to estimate QALYs. To assist those wishing to use these results we provide an online calculator in Excel that provides predicted EQ-5D scores for any user-defined set of WOMAC inputs, age and sex (see Additional file 1).

We find that the stiffness component of WOMAC is relatively unimportant in determining EQ-5D values, consistent with Barton et al. [5] and Grootendorst et al. [7]. Pain and functional disability are much more strongly associated with changes in health utilities but these relationships are different between five classes of patients, identifiable from their disease characteristics. Whether disease was of the hip or the knee did not impact the relationships we identified.

Mitchell et al. [11] recently report a “mapping” study linking to the recently developed ICECAP-0 capabilities measure which, whilst anchored around a 0–1 scale, is not a health utility based instrument. The study only considers linear models on the basis that there are only a small number of observations at the maximum value. Whether the spread of values in the dataset truly represents the spread of severity requires consideration but in any case, the performance of the mixture model approach advocated here in the context of health state utility values should also be considered in the context of other outcome measures such as ICECAP.

Our analysis is based on single sample of Spanish patients receiving a variety of treatments within the broad spectrum of standard practice. In using the results, consideration must be given as to whether this sample includes these for the decision problem at hand and whether the specific treatments under consideration might in some way change the relationship between pain, stiffness, function and health utility.

## Conclusions

EQ-5D can be reliably estimated from WOMAC subscale scores without any systematic bias by using the results presented here.

## Competing interests

The authors declare that they have no competing interests.

## Authors’ contributions

AW and MHA conceived the study, carried out the analyses and wrote the manuscript. AEM acquired the data, contributed to the design and interpretation of results and commented on previous drafts. All authors read and approved the final manuscript.

## Supplementary Material

Additional file 1 EQ-5D calculator. Click here for file
